# Professor Tyndall on the Chemistry of Light—New Experiments and Conclusions

**Published:** 1871-07

**Authors:** 


### PROFESSOR TYNDALL ON THE CHEMISTRY OF LIGHT—NEW EXPERIMENTS AND CONCLUSIONS.

On the 27th of January a paper by Professor Tyndall was communicated to the Royal Society, bearing the title "Action of Rays of High Refrangibility on Gaseous Matter," and he has recently repeated some of his experiments before the Chemical Society. During the last ten years he has conducted a series of investigations in which his principal aim was to render the longest waves of the prismatic spectrum interpreters and expositors of molecular conditions.

Reserving the full discussion of the subject for a future time, it may be stated in a general way (says Professor Tyndall) that all the phenomena of polarization observed in the case of the sky are manifested by the blue actinic clouds. They constitute, to all intents and purposes, pieces of artificial sky, and furnish an experimental demonstration of the constitution of the real one. They moreover exhibit additional phenomena which it would be neither convenient to pursue, nor perhaps possible to detect, in the actual firmament, enabling us to follow the polarization from its first appearance in the barely visible blue to its final extinction, when the particles have become too course to scatter perfectly polarized light.

The principal results of this first part of the inquiry may thus be summed up:

1. The incipient cloud, as long as it continues blue, discharges polarized light in all directions; but the direction of maximum polarization is at right angles to the illuminating beam.

2. As long as the color of the cloud remains distinctly blue the light discharged by it at right angles to the beam is perfectly polarized. It may be entirely quenched by a Nicol's prism, the cloud from which it issues disappearing utterly. Any deviation from the perpendicular direction enables a portion of the light to reach the eye.

3. A plate of tourmaline with its axis parallel to the beam, stops the light; with its axis perpendicular it transmits the light. Hence the direction of vibration of the ether particles all round the beam is perpendicular to the beam.

4. A plate of selenite placed between the Nicol's prism and the cloud, shows the colors of polarized light, and as long as the cloud continues blue these colors are most vivid in the direction perpendicular to the beam. The cloud plays the part of a Nicol's prism.

5. The particles of the incipient cloud are immeasurably small, being at first far beyond the range of the most powerful microscope. But they gradually grow in size, and at a certain period of their growth cease to discharge polarized light. For some time afterward the light that reaches the eye when the Nicol is in its position of minimum transmission is of a magnificent blue color. In the present researches it is called the residual blue.

6. This proves that the ether waves which first feel the influence of the size in the case of the growing particles are the smallest waves of the spectrum. These waves are the first scattered, forming the primary blue; they, also, first escape from polarization and form the residual blue. Neither blue belongs to the matter composing the particles, for this matter may be transparent. The color is solely due to the smallness of the particles.

7. As the actinic cloud grows coarser in texture, the direction of maximum polarization changes from the perpendicular, and incloses an angle more or less acute with the axis of the illuminating beam.

8. In passing from section to section of the same cloud the plane of polarization often undergoes a rotation of 90°.

9. The chemical penetrability of various vapors and mixtures has also been examined, and experiments are described where radiant heat at extraordinary intensity had been brought to bear upon vapors which showed energetic action under the feeblest waves of the spectrum. In no case were the powerful ultra-red rays found competent to produce visible action. They are as powerless here as they are upon the retina.

